# Developing a Charlson Comorbidity Index for the American Indian Population Using the Epidemiologic Data from the Strong Heart Study

**DOI:** 10.21203/rs.3.rs-3369370/v1

**Published:** 2023-09-27

**Authors:** Paul Rogers, Christine Merenda, Richardae Araojo, Christine Lee, Milena Lolic, Ying Zhang, Jessica Reese, Kimberly Malloy, Dong Wang, Wen Zou, Joshua Xu, Elisa Lee

**Affiliations:** National Center for Toxicological Research, Division of Bioinformatics and Biostatistics, U.S. Food and Drug Administration; Office of the Commissioner, Office of Minority Health and Health Equity, U.S. Food and Drug Administration; Office of the Commissioner, Office of Minority Health and Health Equity, U.S. Food and Drug Administration; Office of the Commissioner, Office of Minority Health and Health Equity, U.S. Food and Drug Administration; Office of the Commissioner, Office of Minority Health and Health Equity, U.S. Food and Drug Administration; Department of Biostatistics and Epidemiology, College of Public Health, University of Oklahoma Health Sciences Center; Department of Biostatistics and Epidemiology, College of Public Health, University of Oklahoma Health Sciences Center; Department of Biostatistics and Epidemiology, College of Public Health, University of Oklahoma Health Sciences Center; National Center for Toxicological Research, Division of Bioinformatics and Biostatistics, U.S. Food and Drug Administration; National Center for Toxicological Research, Division of Bioinformatics and Biostatistics, U.S. Food and Drug Administration; National Center for Toxicological Research, Division of Bioinformatics and Biostatistics, U.S. Food and Drug Administration; Department of Biostatistics and Epidemiology, College of Public Health, University of Oklahoma Health Sciences Center

**Keywords:** American Indian, Morbidity, Mortality, Charlson Comorbidity Index

## Abstract

**Background:**

The Charlson Comorbidity Index (CCI) is a frequently used mortality predictor based on a scoring system for the number and type of patient comorbidities health researchers have used since the late 1980s. The initial purpose of the CCI was to classify comorbid conditions, which could alter the risk of patient mortality within a one-year time frame. However, the CCI may not accurately reflect risk among American Indians because they are a small proportion of the U.S. population and possibly lack representation in the original patient cohort. A motivating factor in calibrating a CCI for American Indians is that this population, as a whole, experiences a greater burden of comorbidities, including diabetes mellitus, obesity, cancer, cardiovascular disease, and other chronic health conditions, than the rest of the U.S. population.

**Methods:**

This study attempted to modify the CCI to be specific to the American Indian population utilizing the data from the still ongoing The Strong Heart Study (SHS) - a multi-center population-based longitudinal study of cardiovascular disease among American Indians.

A one-year survival analysis with mortality as the outcome was performed using the SHS morbidity and mortality surveillance data and assessing the impact of comorbidities in terms of hazard ratios with the training cohort. A Kaplan-Meier plot for a subset of the testing cohort was used to compare groups with selected mCCI-AI scores.

**Results:**

A total of 3,038 Phase VI participants from the SHS comprised the study population for whom mortality and morbidity surveillance data were available through December 2019. The weights generated by the SHS participants for myocardial infarction, congestive heart failure, and high blood pressure were greater than Charlson’s original weights. In addition, the weights for liver illness were equivalent to Charlson’s severe form of the disease. Lung cancer had the greatest overall weight derived from a hazard ratio of 8.308.

**Conclusions:**

The mCCI-AI was a statistically significant predictor of one-year mortality, classifying patients into different risk strata *X*^*2*^ (8, N = 1,245) = 30.56 (p = .0002). The mCCI-AI exhibited superior performance over the CCI, able to discriminate between participants who died and those who survived 73% of the time.

## Introduction

The Charlson Comorbidity Index (CCI) is a frequently used mortality predictor based on a scoring system for the number and type of patient comorbidities employed by health researchers since the late 1980s [1]. The initial purpose of the CCI was to classify comorbid conditions, which could alter the risk of patient mortality within a one-year time frame. Mary Charlson, MD pioneered the development of the CCI by weighting 19 different comorbid disorders in a one-year longitudinal study of 559 patients admitted to the New York Hospital-Cornell Medical Center in 1984. The weights were based on the results of a Cox Proportional Hazards survival model that initially included 30 comorbidities for this patient cohort. While specific demographics of the patients in Charlson’s study are unknown, it is known that they were sampled from the local population.

Since its introduction, the CCI has been modified for different medical outcomes and demographic groups. For example, the CCI index has been recalibrated for the population of South Korea; this adjusted index showed a greater mortality prediction than the original CCI [2]. In an international effort to align the CCI weights with advances in disease management and treatment, the index was recalibrated using hospital discharge abstracts from six different countries [3]. The CCI was also validated for specific outcomes such as stroke, head and neck cancers, peritoneal dialysis, and intensive care unit use [4–7]. However, to our knowledge, the CCI has not been recalibrated for any specific racial or ethnic population in the U.S.

Other prediction models and risk-based calculators for hypertension, diabetes, and coronary heart disease (CHD) have been constructed for the American Indian population with epidemiological data. A Cox proportional model with time-varying covariates predicted the likelihood of a non-hypertensive adult American Indian developing hypertension in four years [8]. Similarly, a multivariate logistic regression model predicted the likelihood of a non-diabetic adult American Indian developing diabetes within four years [9]. Gender-specific risk calculators for CHD in a population with a high prevalence of diabetes over 10 years were produced for the American Indian population using a Cox proportional hazards model [10].

This study focuses on calibrating the CCI for the American Indian population using medical information from the Strong Heart Study (SHS), a longitudinal epidemiological study of cardiovascular disease (CVD) among American Indians.

The SHS began in 1988, had multiple phases, consisting of a clinical examination, personal interview, and ongoing mortality and morbidity survey [11]. Participants from 13 different American Indian tribes were recruited from Arizona, Oklahoma, and the Dakotas, employing and enlisting volunteers from each community to promote participation [12]. Figure A1 shows the SHS phases by year and sample size.

Health-related events involving treatment and subsequent causes of mortality were determined by physicians on the SHS Morbidity and Mortality review committees.

A motivating factor in calibrating a CCI for the American Indian population is they experience a greater burden of comorbidities, including diabetes mellitus, obesity, cancer, and cardiovascular disease, than the rest of the US population [13, 14]. A calibrated CCI, specific to the American Indian population, could potentially benefit healthcare providers who serve this community by identifying different levels of risk strata where participants are assigned based on their score. We refer to this modified CCI for the American Indian as the mCCI-AI. We hypothesized that the mCCI-AI instrument created for the American Indian population would:

1. be fundamentally different from the original CCI in terms of assigned comorbidity weights,
2. demonstrate the mCCI-AI to be a significant predictor of one-year mortality, and
3. confirm the mCCI-AI to be granular enough to establish significant risk strata within the American Indian populations.

## Methods

Our study population included all surviving members from Phase VI of the SHS (2014–2018), the most recent publicly available dataset. Data from the five prior phases supplemented the medical history recorded for the Phase VI participants. A one-year time-to-event dataset for this study was constructed from the date of examination in Phase VI. The morbidity and mortality surveillance, and all prior phases, cumulatively provided information on participants’ medical conditions and mortality outcomes. These included 3,038 Phase VI participants and mortality and morbidity surveillance data through December 2019.

The methodology Charlson developed produced a weighted index of mortality based upon the results of a Cox Proportional Hazards model using one year of survival data that included 30 different comorbid diseases. Comorbidities of primary interest in this study were those used in Mary Charlson’s original CCI. These included myocardial infarction, congestive heart failure, peripheral artery disease (PAD), cerebrovascular disease, dementia, chronic pulmonary disease, connective tissue disease, ulcer disease, liver disease, diabetes, hemiplegia, renal disease, diabetes, diabetes with end-organ damage, any tumor, leukemia, lymphoma, solid metastatic tumor, and HIV/AIDS. Participants with these conditions in Phases I through VI were identified using a questionnaire and morbidity and mortality data. According to the SHS data dictionaries and operations manuals, no data was collected during the examination phases on dementia, ulcer disease, hemiplegia, or HIV/AIDS, which were included in the original CCI. The SHS morbidity surveillance results were the sole reports of these conditions. Weights were based on the magnitude of the hazard ratios (HR): conditions with a hazard ratio 1.2 ≤ HR < 1.5 were assigned a weight of 1; those with a hazard ratio 1.5 ≤ HR < 2.5 a weight of 2; conditions with a hazard ratio 2.5 ≤ HR < 3.5 a weight of 3; and those with a hazard ratio greater than six were assigned a weight of 6 [1]. There were no conditions weighted as 4 or 5. The CCI score for an individual was simply the sum of the weights for each condition. For example, in Charlson’s initial assignment of weights, congestive heart failure was assigned a weight of 1 and leukemia a weight of 2. Individuals with these two conditions and no others were given a score of 3. Higher CCI scores indicated a greater likelihood of one-year mortality.

The available SHS participants were divided into training and testing cohorts. Sixty percent of the study sample was randomly allocated to the training cohort, while the remaining 40% was assigned to the testing cohort. The purpose of the training cohort was to generate the hazard ratios used for calculating the mCCI-AI scores with a Cox-Proportional Hazards model. In contrast, the testing cohort would determine whether the mCCI-AI would have any real predictive value for one-year mortality.

One-year survival analysis with mortality as the outcome was performed using the SHS morbidity and mortality surveillance data and assessing the impact of comorbidities in terms of hazard ratios with the training cohort. The level of significance was set to 0.10, as in Charlson’s original study. SHS morbidity and mortality surveillance follow-up extended through December 2019. Survival time was measured in days from the date of the Phase VI examination up to one year from this examination date or to the date of death. Comorbid diseases for the conditions of interest for each participant were coded as a one or zero for present or absent, respectively.

The weights via the training cohort were scored as they were in Charlson’s initial research. Upon completion, each individual in the testing cohort received both an mCCI-AI score based on the results from the training cohort and a CCI score based on the traditional weights. These two scores were then compared regarding their ability to predict one-year mortality. They were included as a single covariate in separate survival models using the test cohort to ascertain if the mCCI-AI or CCI scores were predictive of one-year mortality. Harrell’s C-statistic was adopted to compare the concordance between the two indexes. SAS software version 9.4 was used to perform the analysis and modeling.

## Results

The Phase VI Strong Heart Study consisted of 3,038 individuals, of which 1,142 (37.6%) were males, and 1,896 (62.4%) were females. Table A1 gives the mean age of males and females at 57.4 and 60 years, respectively. The Cox Proportional Hazards model did not control for age and sex, mirroring the original CCI.

The Phase VI questionnaire results and those of the prior five phases were used to construct a longitudinal medical history for each subject. Medical conditions that the study could follow consistently included: myocardial infarction, congestive heart failure, stroke, arthritis, liver disease, PAD, diabetes, renal disease, liquid tumors (leukemia/lymphoma), lung cancer, colon cancer, and high blood pressure. The frequencies and percentages of each of these conditions are given in Table 1 by gender. According to the morbidity and mortality surveillance results, 85 participants died within one year of their SHS Phase VI exam.

Participants had a 60% and 40% probability of selection into the training and testing cohort, respectively. These selection probabilities randomly assigned 1,793 participants to the training cohort and 1,245 to the testing cohort. The training cohort was used to generate the hazard ratios for the various comorbidities within our Cox Proportional Hazards model. The testing cohort participants received both an mCCI-AI and a CCI score based on the results of the statistically significant hazard ratios generated by the survival model with the training cohort.

As study participant relatives were recruited in Phase III, we added a Sandwich Estimator to our Cox Proportional Hazards model to account for the correlation among family members [15]. The results for the training cohort used to develop the hazard ratios for the various medical conditions were roughly the same with and without the Sandwich Estimator, implying negligible correlation.

Charlson’s original work did not mention a check for proportional hazards, an essential requirement of the Cox Proportional Hazards model. Each of our factors from Table 1 was checked for proportional hazards, with stroke and PAD failing this test. As a result, these two conditions were removed from the model; therefore, we could not assess the magnitudes of their hazard ratios. Table 2 contains the results for conditions found to be statistically significant in the survival model. Based on the magnitudes of the hazard ratios, we weighted each condition using the same method as Charlson did in her original research. The weighting for each medical condition is included in Table 3, along with Charlson’s original weights for comparison.

Once we generated the weights for our medical conditions, we calculated both the CCI and mCCI-AI scores for individuals in the testing cohort. The CCI scores ranged from 0 to 9, with 69% of the values at one or lower. The mCCI-AI scores ranged from 0 to 14, with 71 % having scores of 4 or lower. The CCI and mCCI-AI were each placed as a single independent variable in the Cox Proportional Hazards model to determine if either index was predictive of mortality. Both the CCI and mCCI-AI scores were statistically significant predictors of one-year mortality. The results for the CCI were *X*^*2*^(7, N = 1,245) = 28.97 (p = .0001) and for the mCCI-AI *X*^2^ (8, N = 1,245) = 30.56 (p = .0002).

**Figure 1:**
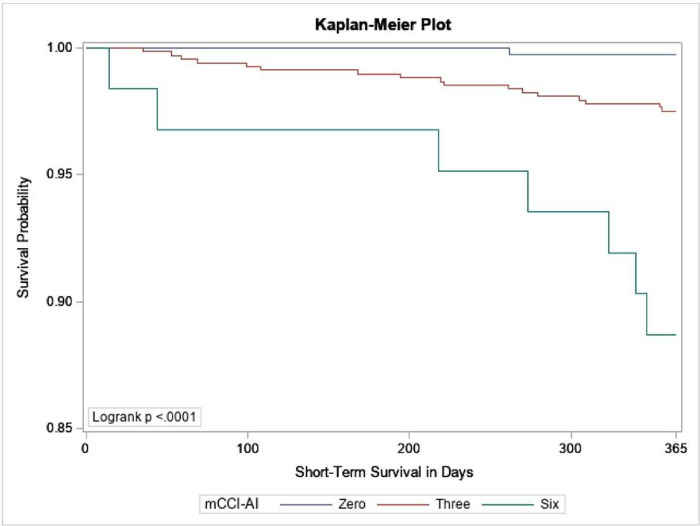
Kaplan-Meier Plot of Short-term Mortality for Participants with mCCI-AI Scores of Zero, Three, and Six.

We used Harrell’s concordance C-statistic to compare the mCCI-AI and CCI scores. This statistic indicates the score’s ability to distinguish between SHS participants that survived and those that died within a year of their Phase VI exam. It was 0.7316 and 0.6597 for the mCCI-AI and CCI scores, respectively. That is, the mCCI-AI could distinguish between participants that died and those that remained living within a year of their Phase VI SHS exam 73% of the time. The CCI, with less discriminatory power, could distinguish survivors from non-survivors only 66% of the time. Last, a Kaplan-Meier plot for a subset of the training cohort was used to compare groups with mCCI-AI scores of zero, three, and six. The Kaplan-Meier survival plot for these three groups is given in Figure 1 and are significantly different from one another according to the logrank test.

## Discussion

As noted in Table 3 above, the SHS study included data on multiple diseases and conditions, some of which were not included in Charlson’s original study and vice versa. For example, Charlson’s study included HIV/AIDS, while this study did not. We covered lung cancer in this study while Charlson ignored this condition, as her test cohort were patients undergoing breast cancer treatment. In addition, two of the included conditions, stroke, and PAD, failed the proportional hazards assumption and were excluded from this study. Still, there were several diseases and conditions common to both studies. This study found that myocardial infarction, congestive heart failure, liver disease, high blood pressure, and lung cancer were significant predictors of one-year mortality in American Indians. Myocardial infarction, congestive heart failure, and high blood pressure were weighted higher in our study than in Charlson’s original study. The weight for the SHS liver disease was the same as that in Charlson’s severe liver disease condition. Last, lung cancer had the greatest weight in this study. We could not compare this condition to Charlson’s as she did not track specific cancers in her original work, but it was equivalent to her findings for solid metastatic tumors.

The observed differences may be due to a real difference in the health of the American Indian population from those of other racial and ethnic populations and the overall U.S. population. Indeed, research has shown higher rates of asthma, type 2 diabetes, obesity, and heart disease among the American Indian populations, both tribally enrolled and non-enrolled, urban and rural [16]. American Indian populations have been found to have low health literacy and distrust of the medical system, so they may not know they need to seek care or may be resistant to seeking care. In addition, emergency medical services were understaffed and under-equipped, reservation health facilities lacked specialty care and, in some instances, basic equipment [17]. This is compounded by poor road conditions, lack of reliable transportation, long distances to health facilities, and extreme distances to hospitals [17]. The picture of the urban American Indian population is more nuanced. The overall health of the American Indian population was fair or poor, regardless of where they resided. Urban-based American Indian populations had higher rates of disability than their rural counterparts but lower rates of hypertension, diabetes, and fewer comorbidities. These personal, cultural, and structural issues may be contributing to the health differences of the American Indian population and be reflected in higher weights in the mCCI-AI.

In addition, there have been advances in medical treatments, technology, and procedures since the 1980s when Charlson’s original study was conducted, but the American Indian populations may not have benefited from these advances. Last, observed differences may be a result of the limited participation of the American Indian population in Charlson’s study conducted in New York in comparison to the Strong Heart Study, where all participants were from the American Indian population [18].

## Conclusion

The mCCI-AI was a statistically significant and better predictor of mortality than the original CCI. This was confirmed by the Kaplan-Meier plot for groups of SHS participants that were assigned mCCI-AI scores of zero, three, and six. Harrell’s concordance statistic demonstrated that the mCCI-AI had more discriminatory power than the original CCI. A tool such as the mCCI-AI allows a more accurate assessment of American Indian participants relative to one-year mortality than could be provided by the original CCI.

Possible future directions include refining the mCCI-AI with more granular electronic health record data. A project of this scope would require the Indian Health Service to give researchers access to its electronic health records system.

### Study Limitations:

The SHS study included data on multiple diseases and conditions, some of which were not included in Charlson’s original study. The SHS data set did not include individuals from every tribe, so the mCCI-AI may not be representative of the total American Indian population.

## Figures and Tables

**Table 1.** SHS medical condition frequencies with percentages in parentheses by gender.

Medical Condition	Population	Male	Female
Myocardial			
Myocardial Infarction	258 (8.5)	127 (11.1)	131 (6.9)
Congestive Heart Failure	163 (5.4)	59 (5.2)	104 (5.5)
Vascular			
Peripheral Artery Disease	215 (7.1)	64 (5.6)	151 (8.0)
Stroke	207(6.8)	73 (6.4)	134 (7.1)
High Blood Pressure	2,030 (66.8)	834 (73.0)	1,196 (63.1)
Connective Tissue Disease			
Arthritis	1,499 (49.3)	491 (43)	1,008 (53.2)
Liver			
Liver Disease	125 (4.1)	53 (4.6)	72 (3.8)
Endocrine			
Diabetes	1,310 (43.1)	477 (41.8)	833 (43.9)
Renal			
Renal Disease	312 (10.3)	119 (10.4)	193 (10.2)
Cancer			
Lung Cancer	19 (0.6)	6 (0.5)	13 (0.7)
Colon Cancer	29 (1.0)	14 (1.2)	15 (0.8)
Leukemia/Lymphoma	18 (0.6)	5 (0.4)	13 (0.7)

**Table 2.** Cox Proportional Hazards model results for statistically significant (alpha = 0.10) factors for Short-Term Mortality with the training cohort.

Variable	Estimate	SE	Chi-Square	p-value	Hazard Ratio
Myocardial Infarction	0.6911	0.3908	3.1260	0.0771	1.996
Congestive Heart Failure	1.0666	0.3909	7.4440	0.0064	2.906
Liver Disease	1.2253	0.4045	9.1729	0.0025	3.405
Lung Cancer	2.1171	0.6622	10.2208	0.0014	8.308
High Blood Pressure	1.0242	0.4002	6.5478	0.0105	2.785

**Table 3.** Comparison of Weighted CCI and mCCI-AI Indices for Short-Term Mortality

Assigned Weights for Comorbidities	Conditions
Charlson's original CCI weights	
1	Myocardial Infarction
	Congestive Heart Failure
	Peripheral Vascular Disease
	Cerebrovascular Disease
	Dementia
	Chronic Pulmonary Disease
	Connective Tissue Disease
	Ulcer Disease
	Mild Liver Disease
	Diabetes
2	Hemiplegia
	Moderate or Severe Renal Disease
	Diabetes with End Organ Damage
	Any Tumor
	Leukemia
	Lymphoma
3	Moderate or Severe Liver Disease
6	Metastatic Solid Tumor
	HIV/AIDS
Strong Heart Study Weights	
2	Myocardial Infarction
3	Congestive Heart Failure
	Liver Disease
	High Blood Pressure
6	Lung Cancer

## Data Availability

The data for this project was provided by the Strong Heart Study (https://strongheartstudy.org/).
